# Enhancing Performance and Quality of Life in Lower Limb Amputees: Physical Activity, a Valuable Tool—A Scoping Review

**DOI:** 10.3390/healthcare14020253

**Published:** 2026-01-20

**Authors:** Federica Delbello, Leonardo Zullo, Andrea Giacomini, Emiliana Bizzarini

**Affiliations:** 1 Spinal Unit, Department of Rehabilitation, Physical Medicine and Rehabilitation Institute, via Gervasutta 48, 33100 Udine, Italy; federica.delbello@asufc.sanita.fvg.it (F.D.); leonardo.zullo@asufc.sanita.fvg.it (L.Z.); 2 School of Physiotherapy, University of Udine, via Gervasutta 48, 33100 Udine, Italy; andrea.giacomini1997@gmail.com

**Keywords:** amputation, sports activities, personalized training, health-promoting behaviors

## Abstract

Background/Objectives: Lower limb amputation (LLA) negatively affects the physical and psychological health of individuals, leading to a lower quality of life and sedentary lifestyle. The objective of this scoping review is to search for evidence regarding physical activity interventions in individuals with LLA, investigating improvements in specific outcomes related to quality of life and performance. Methods: PRISMA guidelines—extension for scoping reviews—were used to structure the study. The research was conducted between 26 July 2023 and 30 September 2023; it was structured by defining two PICO questions (P = amputation, I = physical exercise, O1 = quality of life, and O2 = performance) through Pubmed, Cochrane, and Pedro databases. The study included subjects with LLA of any etiology, in prosthetic or pre-prosthetic phase, practicing non-competitive physical activity. The results were then subjected to both qualitative and quantitative analysis. Results: Of the 615 studies identified, 18 were included in the review. They consisted of 6 systematic reviews (SR), 5 RCTs, 4 case–control studies, 1 case report (CR), and 2 cross-sectional (CS). Physical activity (PA) interventions were extremely heterogeneous and were, therefore, categorized into 6 modalities: surveys were the most reported strategies (57%), followed by personalized training (23%), strength training (13%), endurance training (13%), combined training (2%), and gait training (5%). Due to the heterogeneity of the studies, the variety of interventions proposed and the different outcomes registered, there is no evidence that one approach is more effective than another, while each group showed benefits on different specific outcomes. In total, five outcome categories were identified: quality of life was the most frequently analysed (42%), followed by cardiovascular fitness (20%), muscular fitness (14%), gait parameters (13%), functionality and disability (11%). Conclusions: PA represents a valuable strategy for improving performance and quality of life in individuals with LLA, offering a variety of interventions. Although there is no evidence that one strategy is better than the others, each activity has proven to be effective on specific outcomes, therefore, the choice must depend on the patient’s necessities. The preferred option should be the personalization of the training according to individual needs, coupled with long-term planning and remote monitoring. Creating meeting places and supporting occasions for sports activities could be a valid option. Further research could help to clarify the benefits of such interventions and enhance the understanding of how to optimize the management of LLA patients.

## 1. Introduction

### 1.1. Benefits of Physical Activity

PA is defined by the World Health Organization (WHO) as “any bodily movement produced by skeletal muscles that requires energy expenditure”, making it a broad definition that includes not only sports and exercise but also activities of daily living. The importance of an active lifestyle that reduces sedentary behavior and promotes increasing levels of PA, adapting form, manner, and intensity levels according to individual conditions such as age, psychophysical condition, and socio-economic level, is now well established [1,2,3,4,5,6]. On the contrary, sedentary behavior represents the fourth highest global risk factor for mortality, promoting the onset of chronic diseases [4,5,6,7,8].

The latest WHO guidelines from 2020 provide global recommendations for all age groups on the levels of PA and the limitations on sedentary behavior to be adopted. The aim is to raise awareness among the population about adopting an active and healthy lifestyle. More specifically, the guidelines state that adults and older individuals should achieve 150–300 min of moderate-intensity PA per week or 75–150 min of vigorous-intensity physical activity, or a combination of the two, combined with muscle-strengthening exercises at least two times a week. Despite the WHO guidelines that describe PA as a need of the whole population, there are no indications for categories of patients like LLAs [1].

It is known that higher physical activity levels in adults are positively associated with better mental and physical health-related quality of life (HRQoL); regular sports activities improve psychological well-being, self-confidence, and coping behavior. Moreover, an active lifestyle can have positive effects on chronic illnesses, improving comorbidities (diabetes, cardiovascular diseases) and secondary conditions; DM and cardiovascular diseases take benefit from regular physical activity due to improved metabolic regulation, enhanced insulin sensitivity and a better quality of life [8,9,10].

### 1.2. Physical Activity, Social Participation, and Quality of Life in Amputees

It is known that an amputation can have a negative impact on the physical and psychological parameters of patients, limiting mobility and social participation [11,12,13,14]. Additionally, amputees are prone to developing secondary complications such as pain (nociceptive or neuropathic, including phantom limb pain), overloading of the intact limb, prosthetic misalignment, osteopenia, or low back pain (LBP) [11,12,13,14,15,16,17,18]. From a social and participation perspective, individuals with amputation often feel excluded and classified in a special category, different from able-bodied individuals; this can lead to depressive syndromes and increasing anxiety, especially in the first post-amputation two years [1,2,3,4,5,6,7,18,19].

In general, an LLA predisposes individuals to sedentary behavior due to significant ambulatory difficulties, much higher energy expenditure during walking and higher oxygen consumption compared to able-bodied individuals. The main obstacles to participate in physical activities or sports include pain, lack of specifically organized programs for individuals with LLA, embarrassment, and unawareness about existing sports facilities [19]. The quality of life (QoL) for these individuals, as well as independence and participation in social activities, is primarily influenced by the ability to walk with a prosthesis. A healthy and active lifestyle positively affects ambulatory abilities (such as speed, time spent walking during the day, and distance covered) in people with LLA, thus improving the ability to participate in physical activities and, overall, the QoL [1,2,3,4,5,6,7,8].

Although it is known that lifestyle has a fundamental role in vascular diseases, there are only a few studies considering its importance in LLA patients [2,14,20,21,22,23,24].

The last decades have shown a shift in statistics regarding etiology. Due to a higher attention to prevention and to progresses in medical care, traumatic amputations have significantly decreased to a prevalence of 6%, while vascular diseases have become the leading cause of amputation in Western European regions and the United States, with a prevalence of 90% [12]. This is partly due to the increasing age of the population and the consequently rising incidence of diabetes in older individuals [13].

The shift from traumatic to vascular etiology has implications on rehabilitation, since chronic conditions require a complex multidimensional intervention that should comprehend strategies for preventing the worsening of the illness, limiting secondary disorders, and managing comorbidities.

Although it is known that lifestyle has a fundamental role in vascular diseases, the benefits of PA in LLA patients are poorly cited in the literature; the field is characterized by paucity of studies and a lack of knowledge regarding the effects that an active lifestyle could have on this patients [2,14,20,21,22,23,24].

### 1.3. Objectives of the Review

It is known that life after LLA can be significantly changed with a severe impact on QoL. Standard of living in amputees depends on the ability to walk with a prosthesis, therefore, it is important to find strategies for enhancing their ambulatory capacities. Despite its fundamental role in prevention, QoL, and physical fitness in the healthy population, there is a lack of knowledge regarding physical activity interventions among amputees. The paucity of studies is associated with an extreme heterogeneity due to the variety of interventions and outcomes considered. It follows that there is a lack of consensus in this field, with the need for further investigation; moreover, despite its fundamental role in prevention, physical activity is not always considered in rehabilitation and follow-up protocols.

The objective of this study is to conduct a review regarding the practice of physical exercise and the benefits it can provide in individuals with LLA in terms of walking performance and QoL, aiming to find objective and easily administered information that can be recommended to this population, not only during the rehabilitation program but also in the long term.

## 2. Materials and Methods

The research protocol was drafted using the Preferred Reporting Items for Systematic reviews and Meta-Analyses guidelines—extension for scoping reviews (see Appendix B for the PRISMA checklist). The final protocol was registered with the Open Science Framework (http://osf.io/h5v3a accessed on 12 December 2025).

Two studies were conducted to address different questions regarding the benefits of PA in individuals with LLA. The first study focused on the improvement of QoL, defined as an increase in well-being perception and a reduction in complications or secondary damages, while the second string answered the question of how PA impacts parameters like cardiovascular fitness, muscle strength, gait kinetics and kinematics, and overall performance.

### 2.1. Search Strategy

For both studies, conducted simultaneously from 26 July 2023 to 30 September 2023, Pubmed, Cochrane, and Pedro databases were used.

The Pubmed and Cochrane search was structured on the PICO method; since the aim of the study was to analyse the effect of PA on two different outcomes (quality of life and gait performance), the Authors structured two PICOs and two distinct strings: (((((amputees [MeSH Terms]) OR (amputation [MeSH Terms])) OR (lower limb amputation [Title/Abstract])) OR (lower extremity amputation [Title/Abstract])) AND (((((((exercise [MeSH Terms]) OR (exercise [Title/Abstract])) OR (sport [Title/Abstract])) OR (training [Title/Abstract])) OR (physical activity [Title/Abstract])) OR (aerobic exercise [MeSH Terms])) OR (activity, physical [MeSH Terms]))) AND (((quality of life [MeSH Terms]) OR (quality of life [Title/Abstract])) OR (SF 36 [Title/Abstract])).

The second string was structured as follows: (((((amputees [MeSH Terms]) OR (amputation [MeSH Terms])) OR (lower limb amputation [Title/Abstract])) OR (lower extremity amputation [Title/Abstract])) AND (((((((exercise [MeSH Terms]) OR (exercise [Title/Abstract])) OR (sport [Title/Abstract])) OR (training [Title/Abstract])) OR (physical activity [Title/Abstract])) OR (aerobic exercise [MeSH Terms])) OR (activity, physical [MeSH Terms]))) AND (((((((quality of walking [Title/Abstract]) OR (endurance [Title/Abstract])) OR (cardiorespiratory fitness [Title/Abstract])) OR (cost of walking [Title/Abstract])) OR (6 min walking test [Title/Abstract])) OR (gait analysis [MeSH Terms])) OR (gait analysis [Title/Abstract])).

With the Pedro database, three simple searches were conducted: (1) “exercise lower limb amput*,” (2) “amput* physical activity,” (3) “amput* exercise”.

### 2.2. Eligibility Criteria

The “Preferred Reported Items for Systematic Reviews (PRISMA) guidelines—extension for scoping reviews” were used as a reference for reporting.

The PECOS framework (Population, Exposure, Comparison, Outcomes, Study design) was used to structure the eligibility criteria for this review:

- Subjects with LLA, any etiology, prosthetic or pre-prosthetic phase; male and female individuals, aged > 18 years (population);
- Non-competitive physical activity and sports practice (exposure);
- Studies including or not a comparison group (comparison);
- Since the aim of the present review is to map the breadth of evidence regarding the field and the topic is characterized by a paucity of studies in literature, the Authors decided to include all clinical and secondary studies, published between 2013 and 2023 (last 10 years); in order to avoid bias due to incorrect translations, the Authors chose to select only the studies written in English or Italian (study design).

The exclusion criteria were:

- Competitive sports practice: high level performances often require more technical prosthetic elements, which have a significant effect on physical performance but are not always accessible due to the expensive costs; for this reason, the Authors decided to exclude them from the study;
- Association to other therapies (“phantom mirror therapy”, physical therapies, virtual reality, “mental imaging”, cryotherapy, etc.); in order to avoid bias, programs that associated PA to specific physiotherapy treatments were excluded;
- Articles ongoing, not yet completed.

### 2.3. Data Extraction

Data extraction was performed independently by two authors (AG, FD). An opinion from a third author (LZ) was sought in cases of uncertainty. The information extracted from each article included the year of publication, study design, type of population (level of amputation), etiology, population age (where indicated), interventions on the sample group and any control group, measured outcomes, and the population size (when indicated). The results were sorted into categories and subjected to quantitative and qualitative analysis. Data charting was performed with Excel.

### 2.4. Quality Appraisal

Quality assessment of the included studies was performed independently by two authors (AG, FD). An opinion from a third author (LZ) was sought in cases of uncertainty.

The AMSTAR 2 checklist was adopted for the quality assessment of systematic reviews, while JADAD score was used for RCT quality appraisal; observational studies were assessed with Newcastle-Ottawa scale (case-control), NIH Quality Assessment Tool (cross-sectional), or NTACT Quality checklist (case series).

Assessment tools were used to grade the studies, and then a summary of the quality of the literature included was performed.

## 3. Results

### 3.1. Study Selection

**Figure 1:**
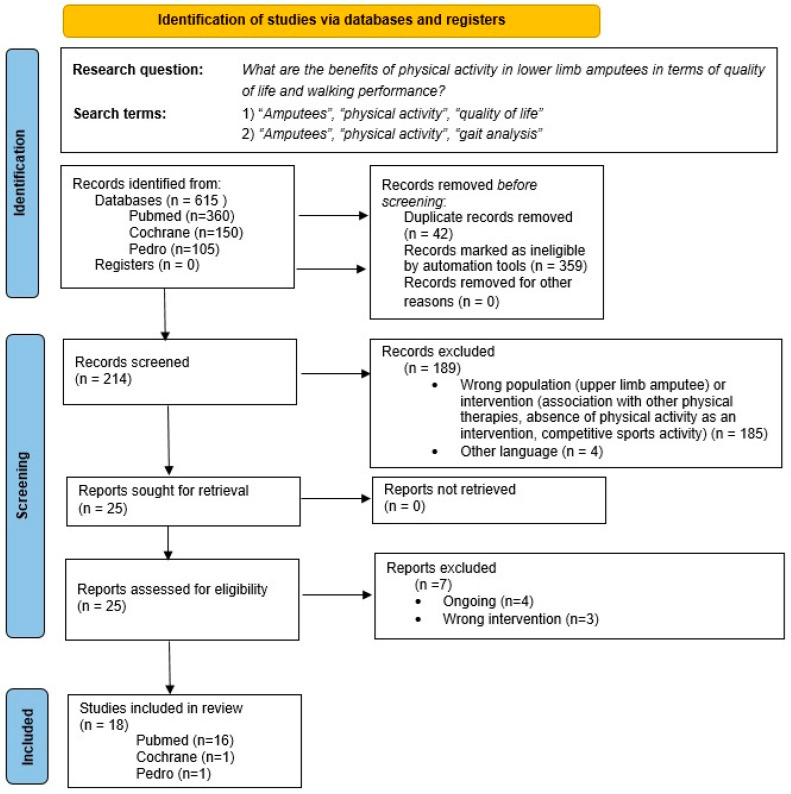
PRISMA flow diagram illustrating the process of identification, screening, and inclusion of the studies.

The research identified a total of 615 studies (360 from Pubmed, 150 from Cochrane, and 105 from Pedro). An initial screening was conducted using filters provided by the three databases, “10 Years Publication” and “Article Type” (“Case Reports”, “Clinical Study”, “Clinical Trial”, “Controlled Clinical trial”, “Meta-analysis”, “Observational study”, “Randomized Controlled Trial”, “Review”, “Scoping Review”, “Systematic Review”), excluding 359 articles. Of the remaining 256 results, duplicates (n = 42) and studies which did not meet the inclusion criteria (n = 189) were removed after Title/Abstract screening: 185 of them were excluded because of wrong intervention (absence of PA, competitive sports practice, application of other physical therapies) or wrong population (upper limb amputations or non-amputated individuals), while four of them were written in another language. Subsequently, through full text screening, seven more articles were excluded; three of them were excluded because of wrong intervention (absence of PA, use of other therapies), and the other four were classified as “ongoing”. In total, 18 studies were included in this review: 16 from Pubmed, 1 from Cochrane, and 1 from Pedro. All 18 articles underwent qualitative analysis, and 16 were included in the quantitative analysis (see Figure 1).

**Figure 2:**
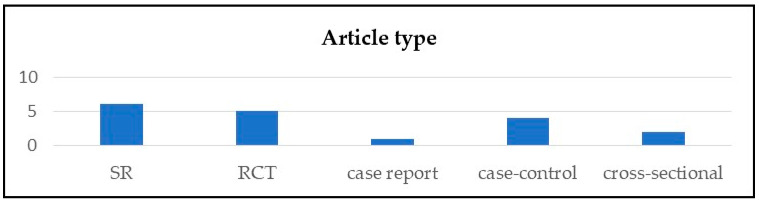
Article type distribution.

**Figure 3:**
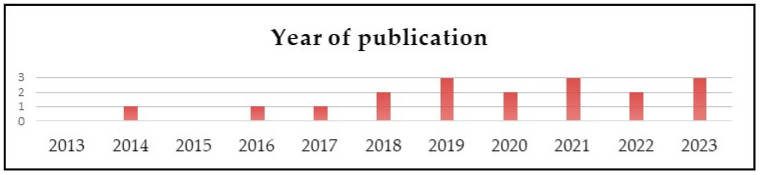
Publication year.

The 18 studies consisted of 6 systematic reviews (SR), 5 randomized controlled trials (RCT), 4 case–control studies, 1 case report (CR), and 2 cross-sectional (CS), published between 2014 and 2023 (Figure 2 and Figure 3).

**Figure 4:**
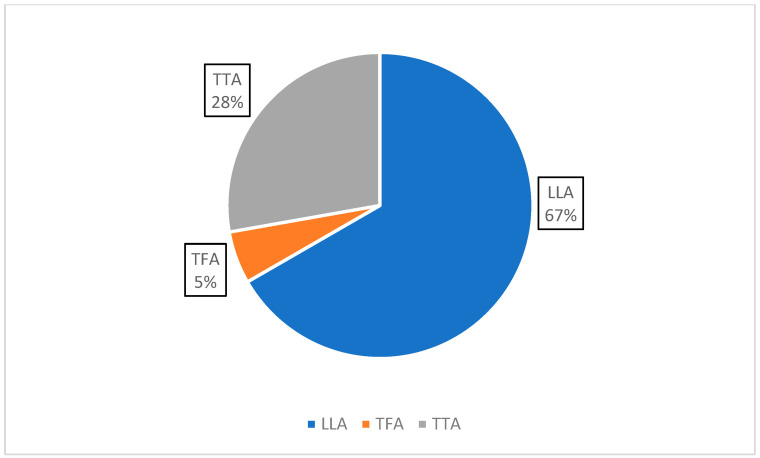
Amputation level.

**Figure 5:**
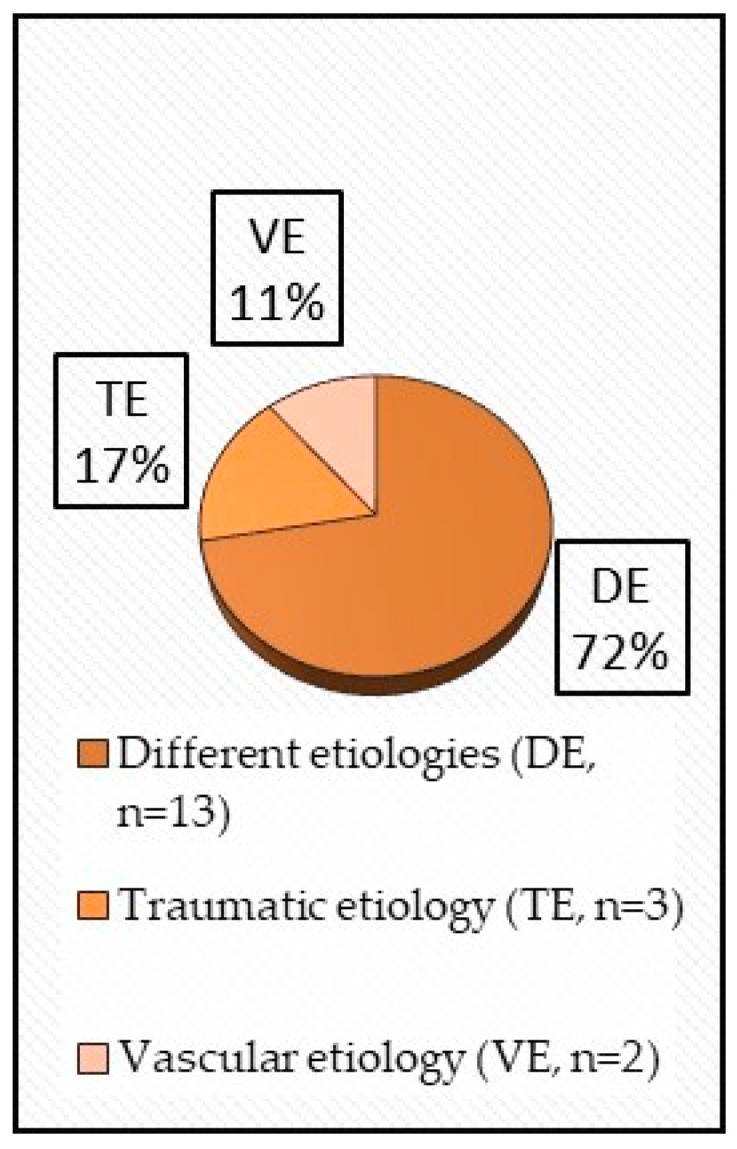
Amputation etiology.

The amputation levels comprehended mixed LLA (12 studies), trans-tibial amputation (TTA) (5 studies), and trans-femoral amputation (TFA) (1 study), while etiology was mixed (13 studies), traumatic (3), and vascular (2) (Figure 4 and Figure 5).

### 3.2. Intervention Categories

**Figure 6:**
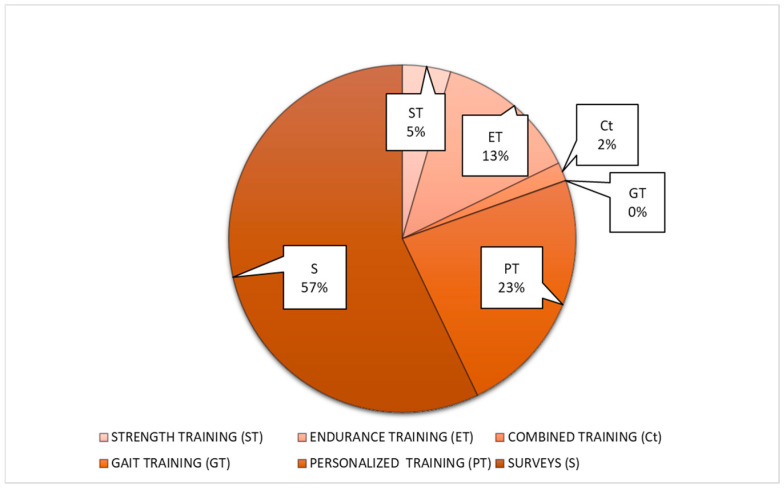
Intervention categories distribution.

The PA interventions administered to individuals in the studies were heterogeneous. To make them more clear and clinically useful, they were grouped into 6 categories: strength training (ST), endurance training (ET), combined training (Ct), gait training (GT), personalized training (PT), and surveys for promoting healthy lifestyle (S) (Figure 6, Table 1).

Each category represents a different approach with different outcomes and clinical relevance.

1. Strength Training: This category includes articles proposing exercises to train muscle strength, such as weightlifting, elastic band exercises, bodyweight exercises, machine exercises, or isokinetic exercises. This category comprises one review and two RCT [24,25,26]. The review demonstrates that a population of individuals with LLA and LBP undergoing strength training benefits significantly from this type of intervention in terms of improving gait parameters, muscle fitness (increase in strength and cross-sectional area of dorsal and core muscles), and, most importantly, QoL (improvement in Short Form Health Survey 36 (SF 36) scores, Oswestry Disability Index (ODI), Roland Morris Disability Questionnaire (RMDQ), and reduction in LBP secondary to amputation) [24]. An RCT shows that daily strength training for individuals with vascular LLA significantly improves QoL outcomes (but not functional and disability parameters) three months post-amputation compared to a control group receiving conventional treatment. However, at six months post-amputation, the same outcomes do not show significant improvements (Barthel Index (BI), Participation Scale, Timed Up and Go (TUG) test, Modified Locomotor Capabilities (LCI)), suggesting short-term benefits; only the EuroQoL 5D scale maintains improvements at six months [25]. The last RCT claims that strength training focusing on hip abductors and extensors performed twice a week for eight weeks by individuals with TFA significantly increases strength and functionality levels (TUG test, 2-Minute Walk test (2MWT), Activities-specific Balance Confidence (ABC) questionnaire [26].
2. Endurance Training: Interventions in this category focus on increasing aerobic capacity, and the main training modalities include the use of the treadmill, cycle ergometer (with only the intact lower limb or with the prosthesis), stationary bike, arm ergometer, rowing machine, and elliptical. Two controlled trials (CT), one RCT, and one SR are included in this category [27,28,29,30]. The two CT consist, respectively, of the administration of three aerobic tests (walking, cycle ergometer, and elliptical) and an aerobic test on the cycle ergometer for 60 min. The first study analyses knee biomechanics in the three tests, showing that the use of the cycle ergometer is the preferred option for TTA patients in terms of reducing secondary damage and preventing osteoarthritis. The second article indicates that, in patients with LLA, maximum oxygen consumption (VO_2_max) and body temperature parameters do not differ from the control group, but they show higher levels of sweating and dehydration [27,28]. An RCT demonstrates that a population of individuals with TFA and TTA with vascular etiology undergoing four weeks of aerobic training three times a week significantly improves VO_2_max values (TFA and TTA) and 6-Minute Walk test (6MWT) outcomes (TTA) [29]. On the other hand, an SR shows that cardiovascular parameters of LLA patients, measured after six weeks of training, significantly improve regardless of the type of aerobic exercise proposed (arm ergometer, cycle ergometer, rowing machine, or combinations). However, the study indicates that a combination of sustained aerobic exercise on the cycle ergometer at a percentage ≥ 50% VO_2_max and on the arm ergometer at a power of 30 Watts is the preferred option to achieve the most significant improvements for effective walking [30].
  Any type of aerobic training (treadmill, arm ergometer, cycle ergometer, or stationary bike, or other modalities) significantly improves cardiovascular parameters, particularly VO_2_max, in individuals with LLA. However, the choice of modality may be dictated by the goals and individual capabilities: for example, cycling training not only improves cardiovascular fitness but also helps reduce the risk of knee osteoarthritis in individuals with TTA; on the other side, to gain an effective ambulation (excluding treadmill training), the preferred solution would be a combination of exercises sustained on the cycle ergometer at medium–high intensity (≥50% VO_2_max) and the arm ergometer (power = 30 W).
  There are no significant differences in VO_2_max and body temperature values between a population with LLA and an able-bodied population; nevertheless, post-aerobic exercise sweating and dehydration increase have been observed in amputated individuals, suggesting that amputees need to assume more fluids to cope with increased dehydration [27,28,29,30]. It follows that hydration status and environmental conditions such as temperature and humidity can represent confounder factors.
3. Combined Training: This category involves a combination of aerobic and strength exercises in different modalities and proportions. Two studies are included in this category [31,32]. A CT shows that eight weeks of concurrent training (a combination of aerobic and strength exercises) are effective in improving cardiovascular (VO_2_max, blood pressure (BP)) and muscular (strength in knee flexor and extensor muscles) parameters compared to conventional training in the control group [31]. The other study (RCT) also submits a population with TTA to eight weeks of concurrent training, demonstrating significant improvements in vascular and strength parameters. Additionally, scores related to the TUG test, Sit-to-Stand (STS) test, Stair Climb test (SCT), and postural balance were investigated and increased after the intervention [32].
  Medium to long-term training programs such as concurrent training in individuals with TTA significantly impact the improvement of cardiovascular and muscular fitness, the reduction of functional and metabolic deficits, and the increase in QoL. This includes a reduction in the risk of chronic diseases and protection from secondary damage. It can be inferred that combined training is a valid and comprehensive training method to improve most of the outcomes of interest. However, further studies on combined training with larger sample sizes would provide more evidence [31,32].
4. Gait Training: In this group, interventions primarily focus on walking abilities and gait quality, including exercises for muscle reinforcement, static and dynamic balance, and walking exercises using parallel bars, treadmill, or free walking. This category includes two studies [33,34]: an SR indicates that combined strength, balance, and walking training are effective in improving gait kinematics outcomes in a population of individuals with LLA in subacute or chronic rehabilitation, while a CR shows that a subject with traumatic TTA undergoing four months of functional walking training (Adaptive Training for an Assist Device, ATAD) achieves better results such as gait kinematics, functionality (K level: K0 to K3), and QoL (SF 36 and reduction in contracture of the knee flexor muscles) [33,34].
  From these interventions, it is evident that positive effects are certainly observed regarding walking parameters, and outcomes related to functionality and QoL also benefit from this type of intervention.
5. Personalized Training: This group includes various intervention modalities that cannot be classified into the previous categories. The trainings involve different combinations of strength, endurance, balance, mobility, and walking exercises personalized according to the characteristics and abilities of individuals. Although this category could seem to overlap the “combined training” group, it is focused on the customization of the program and based on the characteristics of the patient. For this category, two SR and two RCT are included [8,9,35,36]. The two SR present improvements in almost all outcome categories; both studies show that combinations of different types of exercises or even just lifestyle interventions for individuals with LLA, significantly improve cardiovascular and muscular fitness values, gait biomechanics, functionality, and QoL. The main outcome measures investigated and showed significant improvement include VO_2_max, anaerobic threshold (AT), walking energy cost, strength of different muscle groups, 2MWT, Falls Incidence (FI), and gait parameters. No significant improvements were found regarding the TUG test and the LCI scale. The two RCT, finally, propose personalized trainings as intervention, both lasting 12 weeks. The first article analyzes and demonstrates improvements in functionality and disability (Sensory Organization test (SOT), Motor Control test (MCT)), but no improvement in the ABC questionnaire), while the other illustrates significant improvements in the QoL of patients (FI) and gait parameters. It also includes personalised home exercises, suggesting they should be implemented as a method to reduce falls while improving walking ability. In both studies, the first group receives a semi-structured personalization, where a standard program is associated with specific home exercises based on the characteristics of the patient; the semi-structured program permits to associate a standard treatment with a more tailored one. The control group receives standard care in both articles.
  The choice of exercises to include depends on the needs of every person, customizing the training according to different physical and psychosocial abilities. It is important to emphasize that these training programs include various types of interventions but are individualized for each subject. Consequently, they have shown an overall positive effect on all outcome categories.
6. Promoting and monitoring of lifestyle
  This category includes three studies [10,37,38]. There are no direct PA interventions, but questionnaires are administered to the population to monitor QoL and participation in PA. One SR and two OS are included, and these studies show that scores related to the International Physical Activity Questionnaire (IPAQ), SF 36, and World Health Organization Quality of Life (WHOQoL) are significantly lower in population with LLA (two studies with traumatic etiology and one with mixed etiology) compared to the non-amputated population. Furthermore, elderly individuals report lower levels than the adult population, especially regarding the “physical function” and “pain” categories.
  The surveys underlined that the population with LLA reports significantly lower QoL scores compared to able-bodied individuals, especially when dealing with “physical functionality” and “pain” items in older patients; it suggests that a population with LLA needs personalized intervention measures to promote an active lifestyle, improve its quality, and above all, monitor its maintenance over time.

**Table 1:** Intervention categories.

Category	Main Modalities	Articles	n
Strenght training (ST)	dumbbell or barbell;	Godlwana et al. [25];	171
	elastic band;	Pauley et al. [26].	
	bodyweight;		
	gym machines;		
	isokinetic.		
Endurance training (ET)	treadmill;	Orekhov et al. [27];	506
	arm ergometry;	Burger H. et al. [29];	
	lower limb ergometry or bike;	Fukuhara et al. [28];	
	elliptical;	Klenow et al. [30].	
	exercises on hand-wheel;		
	rowing machine.		
Combined training (Ct)	Combination of strength and endurance exercises, in different modalities.	Grecco et al. [31] Grecco et al. [32]	65
Gait training (GT)	Propaedeutics and exercises of:	Kim et al. [34]	1
	static and dynamic balance;		
	strength;		
	gait;		
Personalized training (PT)	Personalized combination of:	Van Helm et al. [9]	884
	strength;	Schafer A. et al. [35]	
	endurance;	Bouzas et al. [8]	
	balance;	Schafer A. et al. [36]	
	flexibility;		
	gait.		
Surveys (S)	Assessment of QoL, Functionality and Physical Activity levels in LLA populations.	De Melo et al. [37]	2168
		Uçkun et al. [38]	
		Christiansen et al. [10]	

### 3.3. Outcome Categories

**Figure 7:**
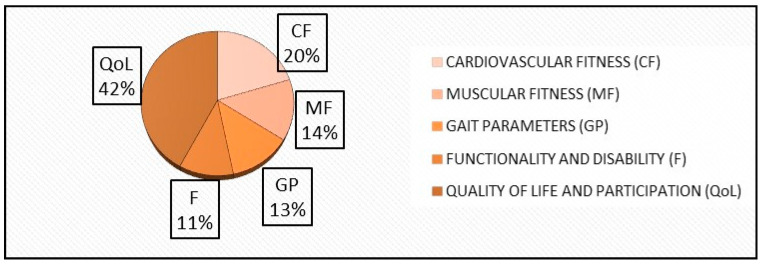
Outcome categories distribution.

As for the interventions, macro-categories with different clinical relevance have been created to group common outcome measures (Figure 7, Table 2).

The outcome categories are:

1. Cardiovascular Fitness (CV), 6 articles included;
2. Muscular Fitness (MF), 6 articles included;
3. Gait Parameters (GP), 6 articles included;
4. Functionality and Disability (F), 7 articles included;
5. Quality of Life and Participation (QoL), 9 articles included.

A synoptic table with a more detailed illustration of interventions and outcomes registered in the studies is available in Appendix A. 

### 3.4. Critical Appraisal

The methodological quality of the included studies is shown in Table 3.

The overall rate of the included RCT was “good”; all the four case–control papers were considered “good”, while the other three observational studies rated from “fair” to “good”. Among the systematic reviews included, only one gained “high quality”, while the other five obtained a “low” rating.

## 4. Discussion

This review explores the effects of PA in people with LLA, in terms of improving QoL (functionality, social participation, disability level) and physical performance (cardiorespiratory, muscular, and ambulatory). The majority of these studies are trials (50% of the total studies, two-thirds of which are RCT) and SR (28% of the total). A total of 83% of the studies were published after 2018, indicating the current interest in the topic.

The research found a large number of studies, suggesting that the topic is of great interest, but data are very heterogeneous, making it challenging to state whether a specific intervention is superior to another.

The review highlights the diversity of PA approaches for individuals with LLA and suggests that each intervention can have positive impacts depending on the evaluated outcomes. It is important to note that results can be influenced by various factors, including age, amputation etiology, K-Level, type of exercises, as well as their mode and duration, suggesting the need of a tailored approach.

The critical appraisal revealed an overall good quality of the studies, except for the systematic reviews included, five of which with a low-quality level; this might depend on the paucity of data in the literature and to the heterogeneity of the interventions, making it difficult to conduct a proper analysis. For what concerns RCTs and observational studies, they gained an overall good mark on quality assessment (Table 3).

Consistency of the results of the highest quality studies is hard to analyse due to the large variety of interventions ad outcomes registered; however, the use of macro-categories makes it possible to draw some conclusions. In general, the studies share the evidence that endurance activities have positive effects on cardiovascular fitness, especially when combined with strength training [8,28,31,32]; personalized interventions focusing on balance and gait in addition to strength and endurance showed improvements in walking ability, functionality and QoL [27,35]; lastly, PA seems to positively influence QoL, independently from the type of intervention [25,27,36,37].

Overall, these results suggest that physical exercise represents a valuable tool to encourage individuals with LLA, as they exhibit lower levels of PA, QoL, and social participation compared to the non-amputee population. The specific modalities of training should be customized based on individual needs and supervised both in the short- and long-term by health professionals to maximize benefits.

Another finding from the review is that all studies involving muscle strength interventions show benefits in the performance and functionality of individuals with LLA, which, in turn, positively affects their QoL. Aerobic and balance (static and dynamic) training also showed effectiveness in improving certain outcomes in amputees.

A considerable number of interventions have been studied and proposed for individuals with LLA, and even though all approaches bring benefits in specific investigated outcomes, the absolute determination of the optimal mode remains elusive. The choice of intervention type for an individual with LLA, therefore, must be based on individual needs. The customization of exercises and their modalities is crucial to achieving positive results, and each proposal should be preceded by a careful assessment of the user’s psychophysical capabilities, priorities, and socioeconomic possibilities. Factors such as age, K-Level, amputation etiology, and the level of amputation are also of primary importance in intervention planning. In particular, the training program should take into consideration the K-level, since it is deemed a fundamental key indicator of LLA functionality.

In any case, some studies have noted that the benefits of PA may be only short-term; hence, it is crucial to plan long-term training programs to maintain these improvements over time. It would be advisable, furthermore, for long-term training programs to be periodically monitored by a professional to detect performance increases, create proper exercise progressions, and keep motivation high in users. Remote monitoring proposals are cited in the literature [38]. Future research could involve applications that track workout progress for both the user and the professional, through online sharable tools or periodic phone calls. Individuals with LLA cannot have a sedentary lifestyle if they want to have an acceptable QoL, and health professionals must be present to assist amputated individuals in leading an active and inclusive lifestyle.

This review showed that a regular physical activity in amputees has benefits in multiple aspects; some of them are strictly related to physical performance (cardiovascular fitness, muscular fitness, gait quality), while others are more linked to psychological status and well-being (quality of life, functionality and disability). QoL is the main outcome emerging from this study; due to its multifaceted nature, this category is assessed with multiple instruments (mainly questionnaires). All the surveys used in the studies are validated, thus giving more evidence.

### Study Limitations

A primary limitation of this review is the small amount of studies found and the heterogeneity of the interventions described, making it difficult to obtain a consensus. It underlines the need for additional studies with larger sample sizes, with the suggestion to include studies with larger populations, that equally investigate each outcome category for each intervention. Due to the heterogeneity of the interventions and the paucity of studies, this review lacks quantitative synthesis and effects size data. Future research could focus on RCTs in order to minimize the risk of bias and ensure a higher evidence to the topic. Moreover, focusing on studies with high quality would ensure more evidence to the research.

Lastly, future research could focus on K-level, differentiating the interventions based on functional status: this classification plays a fundamental role in the assessment of lower limb amputees and each level indicates a different functional outcome; it follows that health professionals should make a distinction among those levels when structuring a PA interventions, making them more clear and specific.

## 5. Conclusions

This review encompasses articles with various study designs, each proposing different PA interventions for individuals with LLA, and investigates specific outcomes related to QoL and performance. While the results obtained are heterogeneous, they highlight common trends in training proposals and suggest some valid tools to optimize the management of amputee patients.

Considering the evident benefits of sports activity and recognizing that individuals with LLA have lower levels of QoL and social participation compared to the non-amputee population, it is essential to propose, encourage, and ensure the practice of PA, for example, creating dedicated facilities where amputees can engage in PA and increase social participation.

While there are positive results in various categories, it is essential to consider some aspects to improve the management of amputee patients:

- Physical activity interventions comprehend strength training, endurance training, gait training, combined approaches, and personalized exercises; each strategy seems to positively influence QoL, independently from the type of intervention chosen;
- Endurance activities can have positive effects on cardiovascular fitness, especially when combined with strength training;
- Personalized interventions focusing on balance and gait in addition to strength and endurance can improve walking ability, functionality and QoL;
- The choice of approach must depend on the patient’s needs, ensuring the customization of interventions;
- Since many studies highlighted the short-term effect of these activities, planning long-term interventions and remote monitoring strategies should be taken into consideration; creating meeting situations and promoting initiatives to encourage the maintenance of PA could represent a solution.

Additionally, further research could contribute towards a better understanding of the long-term effects of PA interventions in individuals with LLA.

The theme of the utility of sports practice in individuals with LLA has been analyzed in its complexity, and from the heterogeneity of the results, some objective and significant methodological guidelines have been drawn. However, further research could shed light upon the long-term effects of such interventions, to better understand how to optimize the management of this population.

## Figures and Tables

**Table 2:** Outcome categories.

Category	Outcome Measures	Articles	n
Cardiovascular fitness (CF)	VO_2_max;	Grecco et al. [31];	1388
	HR max;	Van Helm et al. [9]	
	AT;	Grecco et al. [32]	
	cost of walking;	Burger H. et al. [29]	
	maximal workload.	Bouzas et al. [8];	
		Klenow et al. [30]	
Muscular fitness (MF)	local strength (hip or knee muscles strength);	Grecco et al. [31];	937
	general strength	Van Helm et al. [9]	
	muscle cross-sectional area.	Grecco et al. [32]	
		Bouzas et al. [8]	
		Pauley et al. [26]	
Gait parameters (GP)	walking speed;	Van Helm et al. [9]	871
	center of pressure trajectory;	Kim et al. [34]	
	step length and stance;	Bouzas et al. [8]	
	double support duration;	Schafer A. et al. [36]	
	IL cadence;		
	IL peak vertical force;		
	IL plantarflexion moment in pre-swing phase;		
	hip and IL ankle power absorption and generation.		
Functionality and disability (F)	2MWT and 6MWT;	Van Helm et al. [9]	757
	LCI scale and Modified LCI scale;	Godlwana et al. [25]	
	BI and Modified BI;	Kim et al. [34]	
	TUG test, SCT, and STS test;	Grecco et al. [32]	
	postural balance;	Burger H. et al. [29]	
	SOT, MCT, ABC and AMP questionnaires.	Schafer A. et al. [35]	
		Pauley et al. [26]	
Quality of life and participation (QoL)	Falls incidence;	Van Helm et al. [9]	2878
	SF 36;	De Melo et al. [37]	
	Euroqol 5D;	Godlwana et al. [25]	
	PEQ;	Orekhov et al. [27]	
	WHOQoL;	Uçkun et al. [38]	
	ODI and RMDQ;	Kim et al. [34]	
	IPAQ;	Christiansen et al. [10]	
	Participation Scale;	Schafer A. et al. [36]	
	pain and secondary effects of LLA (such as low back pain, osteoarthritis or contractures);		
	passive and active mobility.		

**Table 3:** Quality assessment.

	Author	Year	Article Type	Checklist	Quality
1	Kim SB [34]	2017	case report	NTACT Quality checklist	acceptable
2	Orekhov G [27]	2019	case–control	Newcastle–Ottawa scale	good
3	Fukuhara K [28]	2021	case–control	Newcastle–Ottawa scale	good
4	Grecco MV [31]	2023	case–control	Newcastle–Ottawa scale	good
5	Grecco MV [32]	2023	case–control	Newcastle–Ottawa scale	good
6	Melo VHD [37]	2021	cross-sectional	NIH Quality assessment tool	good
7	Caliskan UA [38]	2019	cross-sectional	NIH Quality assessment tool	fair
8	Godlwana L [25]	2019	RCT	JADAD	good
9	Pauley T [26]	2014	RCT	JADAD	good
10	Schafer ZA [35]	2021	RCT	JADAD	good
11	Schafer ZA [36]	2018	RCT	JADAD	good
12	Burger T [29]	2022	RCT	JADAD	good
13	Bouzas S [8]	2020	SR	AMSTAR 2	high
14	Wasser J [24]	2020	SR	AMSTAR 2	very low
15	Klenow T [30]	2017	SR	AMSTAR 2	low
16	Madou E [33]	2024	SR	AMSTAR 2	moderate
17	Van Helm S [9]	2022	SR	AMSTAR 2	low
18	Christensen J [10]	2016	SR	AMSTAR 2	low

## Data Availability

The study does not include new data.

## Appendix A

**Table A1:** Synoptic Table of the Included Studies.

Outcome 1: Quality of Life								
Author	Year	Study	Level of Amputation	Etiology	Age	Intervention	n	Outcome
M.V. Grecco et al. [31]	2023	CC	TTA	DE	18 < age < 50	strength, aerobic exercises	34	VO_2_ max
								lower limb strength
								HR max
								Blood pressure
E. Madou et al. [33]	2023	SR	LLA	DE	>18 years old	Gait, balance and strength training	/	gait
S. Van Helm et al. [9]	2022	SR	LLA	DE	>18 years old	strength, endurance, flexibility, balance and gait exercises	520	VO_2_ max
								body mass
								lower limb strength
								EuroQoL 5D
V.H. De Melo et al. [37]	2021	CS	LLA	DE	18 < age < 59: Adults LLA	surveys	36	IPAQ
					>60: Elderly LLA			WHOQoL
L. Godlwana et al. [25]	2020	RCT	LLA	VE	>18 years old	education, strength exercises	154	EuroQoL 5D
								TUG test
J.G. Wasser et al. [24]	2019	SR	LLA	DE	/	Strength + resistance exercises	/	pain
								core fitness/strength
								gait/kinematics
G. Orekhov et al. [27]	2019	CC	TTA	DE	18 < age < 45	gait, cycling and elliptical training	20	walking speed
								lower limb ROM
								gait
A. Uçkun et al. [38]	2019	CS	TTA	TE	18 < age < 65	surveys	102	IPAQ
								SF-36
S.B. Kim et al. [34]	2017	CR	TTA	TE	53	Balance, strength and gait exercises	1	lower limb passive ROM
								K level
								gait
								SF-36
J. Christensen et al. [10]	2016	SR	LLA	TE	/	surveys	2030	SF-36
								QoL
Outcome 2: Performance								
Author	Year	Study	Level of amputation	Etiology	Age	Intervention	n	Outcome
M.V. Grecco et al. [32]	2023	CC	TTA	DE	18 < age < 50	strength, aerobic exercises	31	lower limb strength
								VO_2_ max
								postural balance
								TUG test
								SCT
								STS
Burger H. et al. [29]	2022	RCT	LLA	VE	Mean: 65 years old	aerobic training on hand-wheel at 70–80% of heart rate reserve	20	VO_2_ max
								6MWT
K. Fukuhara et al. [28]	2021	CC	LLA	DE	Mean: 40.3 years old	Arm ergometer	18	VO_2_ max
								metabolic heat production
								temperature
								body/local sweating
Z.A. Schafer et al. [35]	2021	RCT	LLA	DE	Mean: 61.5 years old	home-based exercise sessions consisting of balance, flexibility, gait endurance and strength training	14	SOT MCT ABC Questionnaire
S. Bouzas et al. [8]	2020	SR	LLA	DE	Mean: 47 years old	strength, flexibility, aerobic, gait, balance and combined interventions.	355	VO_2_ max
								muscular strength
								active flexibility
								gait
								2MWT
								TUG test
								LCI
Z.A. Schafer et al. [36]	2018	RCT	LLA	DE	34 < age < 91	combination of strength, endurance, flexibility, balance, and gait exercises;	15	FI
								walking speed
								gait
T.D. Klenow et al. [30]	2018	SR	LLA	DE	mean: 65.4 years old	ergometer, rowing machine	448	VO_2_ max
								maximal workload (Watt)
T. Pauley et al. [26]	2014	RCT	TFA	DE	> 65 years old	hip strength training	17	TUG test 2MWT muscular strength ABC Questionnaire
					(mean: 67.8 years old)			

Abbreviations: RCT, Randomized Controlled Trial; CC, Case-control study; SR, Systematic Review; CR, Case Report; CS, Cross-sectional study; LLA, Lower Limb Amputation; TTA, Transtibial Amputation; TFA, Transfemoral Amputation; DE, Different Etiology; VE, Vascular Etiology; TE, Traumatic Etiology; IPAQ, International Physical Activity Questionnaire; WHOQoL, Whorld Health Organization Quality of Life; HR max, Maximal Heart Rate; 2MWT, 2 Minutes Walking Test; FI, Falls Incidence; LCI, Locomotor Capability Index; TUG test, Timed Up and Go test; SCT, Stair Climb Test; STS, Sit To Stand; 6MWT, 6 Minutes Walking Test; SOT, Sensory Organization Test; MCT, Motor Control Test; ABC, Activities-specific Balance Confidence.

## Appendix B

**Table A2:** Preferred Reporting Items for Systematic Reviews and Meta-Analyses Extension for Scoping Reviews (PRISMA-ScR) Checklist.

Section	Item	PRISMA-ScR Checklist Item	Reported on Page
TITLE			
Title	1	Identify the report as a scoping review.	1
Abstract			
Structured summary	2	Provide a structured summary that includes (as applicable): background, objectives, eligibility criteria, sources of evidence, charting methods, results, and conclusions that relate to the review questions and objectives.	1
Introduction			
Rationale	3	Describe the rationale for the review in the context of what is already known. Explain why the review questions/objectives lend themselves to a scoping review approach.	2–3
Objectives	4	Provide an explicit statement of the questions and objectives being addressed with reference to their key elements (e.g., population or participants, concepts, and context) or other relevant key elements used to conceptualize the review questions and/or objectives.	2–3
Methods			
Protocol and registration	5	Indicate whether a review protocol exists; state if and where it can be accessed (e.g., a Web address); and if available, provide registration information, including the registration number.	3
Eligibility criteria	6	Specify characteristics of the sources of evidence used as eligibility criteria (e.g., years considered, language, and publication status), and provide a rationale.	3–4
Information sources	7	Describe all information sources in the search (e.g., databases with dates of coverage and contact with authors to identify additional sources), as well as the date the most recent search was executed.	3–4
Search	8	Present the full electronic search strategy for at least 1 database, including any limits used, such that it could be repeated.	3
Selection of sources of evidence	9	State the process for selecting sources of evidence (i.e., screening and eligibility) included in the scoping review.	3–4
Data charting process	10	Describe the methods of charting data from the included sources of evidence (e.g., calibrated forms or forms that have been tested by the team before their use, and whether data charting was done independently or in duplicate) and any processes for obtaining and confirming data from investigators.	4
Data items	11	List and define all variables for which data were sought and any assumptions and simplifications made.	4
Critical appraisal of individual sources of evidence	12	If done, provide a rationale for conducting a critical appraisal of included sources of evidence; describe the methods used and how this information was used in any data synthesis (if appropriate).	4
Synthesis of results	13	Describe the methods of handling and summarizing the data that were charted.	4
Results			
Selection of sources of evidence	14	Give numbers of sources of evidence screened, assessed for eligibility, and included in the review, with reasons for exclusions at each stage, ideally using a flow diagram.	5
Characteristics of sources of evidence	15	For each source of evidence, present characteristics for which data were charted and provide the citations.	6–7
Critical appraisal within sources of evidence	16	If done, present data on critical appraisal of included sources of evidence (see item 12).	11–12
Results of individual sources of evidence	17	For each included source of evidence, present the relevant data that were charted that relate to the review questions and objectives.	6–12
Synthesis of results	18	Summarize and/or present the charting results as they relate to the review questions and objectives.	9–11
Discussion			
Summary of evidence	19	Summarize the main results (including an overview of concepts, themes, and types of evidence available), link to the review questions and objectives, and consider the relevance to key groups.	12–13
Limitations	20	Discuss the limitations of the scoping review process.	13
Conclusions	21	Provide a general interpretation of the results with respect to the review questions and objectives, as well as potential implications and/or next steps.	13–14
Funding			
Funding	22	Describe sources of funding for the included sources of evidence, as well as sources of funding for the scoping review. Describe the role of the funders of the scoping review.	14
